# DNA delivery into plant tissues using carbon dots made from citric acid and β-alanine

**DOI:** 10.3389/fchem.2025.1542504

**Published:** 2025-03-19

**Authors:** Kuber Shivashakarappa, Sureshbabu Marriboina, Zeinab Yadegari, Vikas Reddy Paduri, Ritesh Sachan, Korsi Dumenyo, Ali Taheri

**Affiliations:** 1 Department of Agricultural Science and Engineering, College of Agriculture, Tennessee State University, Nashville, TN, United States; 2 Department of Life and Physical Sciences, Fisk University, Nashville, TN, United States; 3 School of Mechanical and Aerospace Engineering, Oklahoma State University, Stillwater, OK, United States

**Keywords:** DNA delivery, nanoparticle, carbon dots, gene expression, plant transformation

## Abstract

Agriculture and food security face significant challenges due to population growth, climate change, and biotic and abiotic stresses. Enhancing crop productivity and quality through biotechnology is crucial in addressing these challenges. Genome engineering techniques, including gene cassette delivery into plant cells, aim to meet these demands. However, conventional biomolecule delivery methods have limitations such as poor efficacy, low regeneration capability, and potential cell damage. Nanoparticles, known for their success in drug delivery in animals, hold promise as DNA nanocarriers in plant sciences. This study explores the efficacy of carbon dots (CDs), synthesized rapidly and cost-effectively from citric acid monohydrate and β-alanine using a microwave-assisted method, as carriers for plasmid DNA delivery into plant tissues. The detailed characterization of carbon dots, evaluation of their binding ability with plasmid DNA, and phytotoxicity assessments were systematically conducted. The delivery and expression of plasmid DNA were successfully demonstrated in canola leaves via needleless syringe infiltration and in soybean root cells and protoplasts through passive diffusion. Additionally, the particle bombardment method facilitated the efficient delivery of plasmid DNA of varying sizes (4 kb, 11 kb, and 17 kb) into onion epidermal cells, as well as the successful delivery of plasmid DNA containing the GUS reporter gene into soybean embryos, using carbon dots as a binding agent between plasmid DNA and tungsten microcarrier. To our knowledge, this is the first study to report the use of carbon dots as a substitute for spermidine in such applications. Overall, our research presents a rapidly synthesized, cost-effective platform for efficient plasmid DNA delivery, establishing a foundation for using carbon dots as carriers for CRISPR and RNAi constructs in gene knockout and knockdown applications in plant tissues, with a comparison of their transformation efficiency against traditional delivery techniques.

## Introduction

Plant genetic engineering is a recent technological innovation in plant science, serving as a crucial tool for promoting sustainability, synthesizing natural products, and improving the quality and production of agricultural crops (Wang et al., 2016; Si et al., 2016). This technology can help meet the demands of a growing human population despite fluctuating global climatic conditions (Nidhi et al., 2021). In recent decades, there has been notable progress in biotechnology, particularly in the development of genome editing and sequencing technologies. These advancements have the potential to increase secondary metabolite in plants (Lv et al., 2019) and advance plant synthetic biology and bioengineering (Wang et al., 2017). Genetic engineering can be utilized in agriculture to enhance crop productivity and nutritional content. Additionally, genetically engineered crops can possess resistance to herbicides, insects, diseases, and abiotic stresses such as drought and heat (Daniell et al., 1998; Liu et al., 2015; Li et al., 2012; Zhang et al., 2009).

Despite considerable advancements in biotechnology, genetic transformation is considered as a main bottleneck in many plant species. This process involves transferring a foreign gene (known as a transgene) that encodes a desired trait into a plant cell, ultimately introducing the desired trait into a crop (Altpeter et al., 2016). One major obstacle to achieving successful plant genetic transformation is delivering biomolecules into plant cells, which is hindered by presence of the hard and thick cell wall. Agrobacterium-mediated transformation is a commonly employed approach in plant genetic engineering (Herrera-Estrella et al., 1983). However, it is only applicable to a limited range of plant species and lacks the ability to achieve transgene-free editing (Baltes et al., 2017). Gene-gun transformation, also known as biolistic particle transformation, is a delivery technology that can be used for any plant species. Despite its versatility, this method may cause tissue damage and often does not result in efficient integration of the genetic material (Wang Jun et al., 2018). Alternative techniques for delivering biomolecules, such as electroporation, polyethylene glycol (PEG), the pollen tube pathway, and cationic transport, face several drawbacks as well. These include low efficiency, limited regeneration capabilities, and potential harm to cells due to damage and cytotoxicity (Yoo et al., 2007; Ozyigit, 2020; Zeng et al., 1998). Overall, genetic engineering in plant systems has significantly lagged behind the advancements achieved in animal systems. Furthermore, plant genetic engineering lacks efficient techniques that allow the introduction of biomolecules into plant tissues using passive diffusion/cellular uptake without requiring external force, which causes considerable tissue damage, resulting in poor transformation efficiency and low regeneration rates. Therefore, it is essential to establish innovative and effective gene delivery approaches which can overcome the limitations associated with conventional gene delivery techniques and enable the passive transport of various biomolecules into a wider range of plant species. The most commonly used methods to deliver biomolecules into plant tissues using nanoparticles as carriers are syringe infiltration, vacuum infiltration, and cellular uptake or passive diffusion mechanisms. Furthermore, the biolistic gene transfer approach, utilizing a gene gun, is recognized as the fastest and most successful technology for inserting genes into various plant tissues (Maliga and Tungsuchat-Huang, 2014). This method employs gold or tungsten particles, typically ranging from 0.6 to 1.0 μm in diameter, co-precipitated with biomolecules using cationic agents and salts like calcium chloride to form a biomolecule-particle complex (Tian and Seguin, 2004). This complex is subsequently propelled by a gas burst in a gene gun instrument with sufficient force to penetrate the cell wall, efficiently reducing harm to the surrounding plant tissue (Liu et al., 2019). Considerable attention has been given to various phases of the bombardment process, including binding, bombardment, and release, with refined methods now available for numerous plant species (Fadeev et al., 2006; Ueki et al., 2009; Zilony et al., 2013). However, insufficient focus has been placed on the interaction between biomolecules and tungsten or gold particles during the binding and release phases, a crucial step in gene delivery. A pivotal element in both phases is the amine-coated cationic agent, which promotes binding between the biomolecule and the tungsten particle via electrostatic attraction. Currently, spermidine, which contains amine groups, is the only molecule used in the biolistic delivery technique (Hamada et al., 2017; Sawant et al., 2000; Zilony et al., 2013). However, spermidine is challenging to synthesize in a standard laboratory setting and is costly. Thus, it is necessary to investigate alternative molecules that can be synthesized in laboratories and are more cost-effective.

Nanoparticle-mediated gene delivery using a nanoscale particle smaller than 100 nm as carrier for various biomolecules offers a promising solution to the limitations of conventional delivery methods in plants (Yin et al., 2017). Nanoparticles exhibit high biocompatibility with host systems, efficiently bypass cellular membranes, and protect genetic cargo from nuclease degradation (Cunningham et al., 2018). Additionally, they enable the delivery of diverse payloads, facilitate precise targeting through chemical and physical modulation, and allow controlled cargo release. This approach addresses the low regeneration rates associated with traditional techniques by enabling direct germline modification in plant tissues (Zhao et al., 2017). Over the past decades, researchers have utilized various nanocarriers to deliver biomolecules such as plasmid DNA, proteins, siRNA, and dsRNA into a wide range of plant tissues across numerous plant species. Metallic nanocarriers, such as gold nanospheres, have been employed for siRNA delivery, achieving knockdown efficiencies exceeding 80%, while iron oxide clusters have been applied for pollen transformation (Zhang et al., 2022; Wang et al., 2022). Silica-based carriers, including mesoporous silica nanoparticles, have been used for protein delivery into intact plants (Martin-Ortigosa et al., 2014). Additionally, bio-inspired carriers, such as liposomes and vesicles, have demonstrated efficacy in delivering genes and proteins into protoplasts and various plant tissues (Mahmoud et al., 2022; Liu et al., 2020).

Carbon-based nanomaterials, including single-walled and multiwalled carbon nanotubes (CNTs) and CDs, have emerged as highly efficient carriers for the delivery of cargoes into plants due to their tunable physicochemical properties (Demirer et al., 2019b; Wang et al., 2020). These properties enable researchers to track the movement of nanoparticles inside plant tissues, providing real-time insight into the delivery process. To enhance their cargo-carrying capacity, these nanoparticles are often functionalized with various cationic polymers, such as polyethyleneimine (Schwartz et al., 2020; Kwak et al., 2019). Several delivery techniques, including syringe infiltration, vacuum infiltration, spray methods, and passive diffusion, have been employed to introduce a range of cargoes into plant tissues effectively to promote transient gene expression (Table 1). Despite the promising attributes of carbon-based nanomaterials, such as the presence of functional organic groups like amine groups on CDs, which can interact with and hold cargoes on their surface, their direct use in gene delivery systems in plants without the need for additional functionalization with cationic polymers through electrostatic union and their potential to promote stable gene expression remains underexplored. Furthermore, their potential as binding agents between plasmid DNA and microcarriers in particle bombardment techniques, as a replacement for currently used amine-coated molecules, has not yet been investigated. Overall, considering the cost, time, specialized skills, and instrumentation required for synthesizing carbon dots previously utilized for gene delivery in plants along with the potential risks associated with the functionalization of nanomaterials, such as polyethyleneimine and other molecules, due to their high cytotoxicity (Lungwitz et al., 2005), and the requirement to investigate an alternative molecule for spermidine in particle bombardment. There is a need to explore carbon dots that can be synthesized rapidly without requiring functionalization and can effectively deliver genes using various insertion techniques, including particle bombardment. CDs can generally be synthesized using two primary methods: the top-down approach and the bottom-up approach (Bruno et al., 2021). The top-down method involves the fragmentation of graphite compounds to produce CDs, while the bottom-up method typically utilizes microwave-assisted synthesis (Ma et al., 2019). In this bottom-up process, carbon sources such as citric acid or dextrin serve as precursors, and compounds like β-alanine or ethanolamine act as passivating agents. Subjecting this mixture to rapid heating in a microwave at specific power levels and durations promotes the carbonization of the precursor, resulting in fluorescent carbon dots with nitrogen incorporated into their backbone and the distribution of amine groups on the surface due to successful surface passivation facilitated by the passivating agents (Pinto et al., 2023). This method of synthesizing CDs is straightforward, rapid, and offers a one-pot synthesis approach. Notably, CDs synthesized using citric acid and β-alanine via the microwave-assisted method, without functionalization with cationic molecules after synthesis, have demonstrated the ability to bind with various plasmid DNA molecules and successfully deliver them into *E. coli* cells (Pandey et al., 2021). In this study, we report the synthesis of carbon dots using citric acid as a carbon source and β-alanine, a derivative of amino acid, to minimize potential phytotoxicity. The CDs were synthesized using a microwave-assisted method and evaluated for their potential as carriers to deliver plasmid DNA into various plant tissues. We present evidence for their use in delivering reporter genes including green fluorescent protein (GFP), red fluorescent protein (dsRED), β-glucuronidase (GUS), and rubyred into various plant tissues using different gene insertion techniques. These tissues include canola leaves via infiltration, soybean roots and soybean protoplasts through passive diffusion of carbon dots-plasmid complexes, and soybean embryos and onion epidermal cells. Additionally, we demonstrate that carbon dots can deliver plasmids containing reporter genes of up to 17 kb, including CRISPR constructs, into plant tissues. Our work provides a quick and affordable method to synthesize carbon dots and demonstrates their effective use as a gene delivery system for rapid gene expression.

**TABLE 1:** Evidence of carbon-based nanoparticle mediated successful delivery of various cargoes into crop plants using different gene insertion techniques to promote transient gene expression.

Carrier	Species	Targeted tissues	Cargo	Insertion method	Expression type	Reference
PEI functionalized SWCNT	*Nicotiana benthamiana* *Eruca sativa* *Triticum aestivum* *Gossypium hirsutum*	Leaves	Plasmid DNA	Syringe infiltration	Transient	Demirer et al. (2019a)
Chitosan functionalized SWCNT	*Nicotiana* *tabacum* *Eruca* *sativa* *Nasturtium* *officinale* *Spinacia* *oleracea* *Arabidopsis* *thaliana*	Leaves Protoplast	Plasmid DNA	Syringe infiltration Passive diffusion	Transient	Kwak et al. (2019)
PEI functionalized CDs	*Oryza Sativa* *Triticum aestivum* *Phaseolus radiatus*	Leaves Root Calli	Plasmid DNA	Vacuum infiltration Passive diffusion Spray method	Transient	Wang et al. (2020)
PEI functionalized CDs	*Nicotiana benthamiana* *Solanum lycopersicum*	Leaves	siRNA	Spray method	Transient	Schwartz et al. (2020)
PEI functionalized CDs	*Cucumis sativus*	Leaves	dsRNA	Spray method	Transient	Martín et al. (2022)
PEI functionalized SWCNT	*Oryza sativa*	Leaves Embryo	Plasmid DNA and CRISPR construct	Syringe Infiltration Vacuum infiltration	Transient	Dunbar et al. (2022)
PEI functionalized SWCNT	*Arabidopsis thaliana*	Leaves	Plasmid DNA	Spray method	Transient	Santana et al. (2022)
β-cyclodextrin functionalized CDs	*Arabidopsis thaliana*	Leaves	Plasmid DNA and Peptide	Spray method	Transient	Santana et al. (2022)
Amine functionalized carbon dots	*Nicotiana benthamiana*	Leaves	Plasmid DNA	Syringe infiltration	Transient	Liu et al. (2023)
Arginine functionalized SWCNTs	*Nicotiana benthamiana*	Roots	Plasmid DNA	Passive diffusion	Transient	Golestanipour et al. (2018)
PEI functionalized SWCNTs	*Nicotiana benthamiana*	Leaves	Plasmid DNA	Syringe infiltration	Transient	Ali et al. (2022)
Polymer-coated SWCNTs	*Arabidopsis thaliana*	Seedlings	Plasmid DNA	Vacuum infiltration	Transient	Law et al. (2022)
α-aminoisobutyric acid functionalized CNTs	*Arabidopsis thaliana*	Seedlings	Plasmid DNA	Vacuum infiltration	Transient	Law et al. (2024)

## Materials and methods

### Preparation of CDs

**FIGURE 1:**
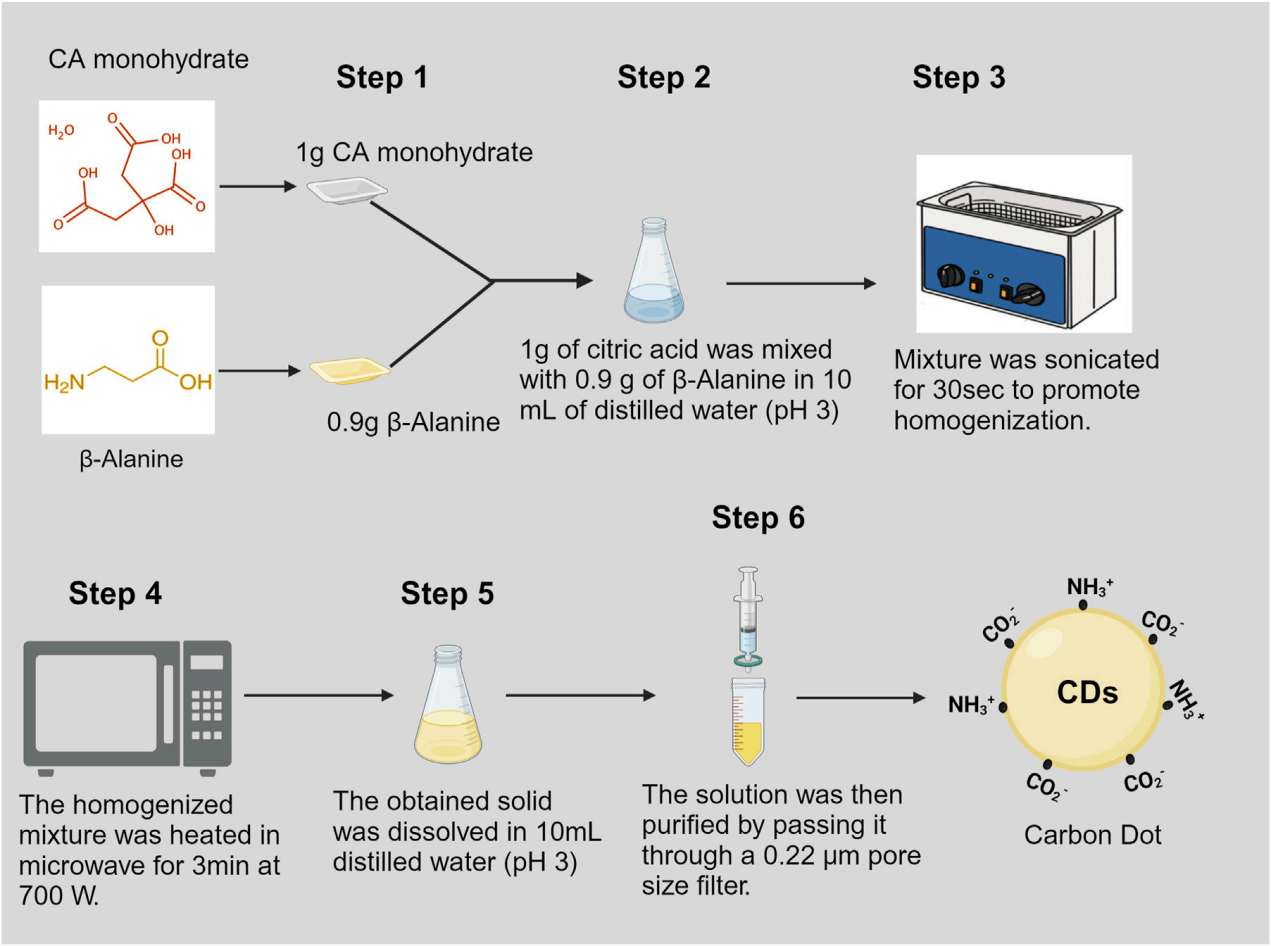
Schematic representation of rapid synthesis of carbon dots.

To synthesize CDs, a solution was prepared by combining citric acid monohydrate (Fisher #A104-500, Pittsburg, PA, United States) and β-alanine (Fisher Cat#AAA166650I, CAS: 107-95-9) in a molar ratio of 1:2. In a conical flask, 1.0 g of citric acid monohydrate and 0.9 g of β-alanine were dissolved in 10 mL of distilled water, with the pH adjusted to 3.0 using hydrochloric acid (HCl). The solution was homogenized using an ultrasonicator (Ultrasonic Cleaner FS30, Fisher Scientific, Pittsburg, PA, United States) and then heated in a commercial microwave oven (Model#JES2251SJ02, GE Appliance, Louisville, KY, United States) set to 70% power for 3 min. This process aims to achieve carbonization and surface passivation, resulting in the formation of a brownish solid. The solid product was then dissolved in 10 mL of distilled water with the pH adjusted to 3.0 using HCl. The carbon dot solution underwent purification through filtration using a 0.22 µm pore size filter, following the method described by Pandey et al. (2021) (Figure 1).

### Characterization and evaluation of DNA binding capacity of CDs

Photoluminescence properties, including absorbance wavelength, excitation wavelength, emission wavelength, and quantum yield of synthesized carbon dots were evaluated using a Synergy H1 Hybrid Multi-Mode Microplate Reader (BioTek, Winooski, VT, United States). The size and morphology of the CDs were examined via high-resolution transmission electron microscopy (HRTEM) using a JEOL JEM-2100 instrument with Bruker EDS and dark-field scanning transmission electron microscopy (STEM) performed using FEI Tecnai G2 Osiris microscope. The functional groups of the carbon dots were analyzed through Fourier-transform infrared (FTIR) spectroscopy using a Perkin Elmer Frontier Infrared Spectrometer (PerkinElmer, Waltham, MA, United States). This instrument was equipped with a liquid nitrogen-cooled mercury cadmium telluride (MCT-A) detector, and the optics compartment was supplied with CO_2_- and H_2_O-free air using a Balston-Parker air purifier. Electrostatic charge measurements for the CDs and CDs/pDNA complex were conducted using a Zetasizer Nano ZS (Malvern Panalytical Inc., Westborough, MA, United States).

Additionally, the experiment was conducted to evaluate the binding capacity between CDs and DNA using a gel electrophoresis assay. Plasmid DNA was mixed with varying concentrations of carbon dots, ensuring thorough homogenization by repeated pipetting. The DNA-CDs complexes were incubated at 37°C for 30 min. After incubation, 2 µL of loading dye was added to the complexes. Gel electrophoresis was then performed using a 1% agarose gel in 1x TBE buffer, with an applied voltage of 85 V for 60 min. The results were visualized under ultraviolet irradiation.

### Evaluation of internalization and phytotoxic effects of CDs in plant tissues

In this experiment, fresh onions were cut into quarters, and sections of onion epidermis (measuring at least 3 × 3 cm) were prepared. These sections were then placed on a water agar medium. To assess whether the CDs could internalize into the onion epidermal cells through passive diffusion mechanism without requiring any external force, 10 µL of the prepared carbon dots were sprinkled onto the surface of each onion epidermis section. The sections were incubated at room temperature for 20 min for internalization analysis. After the incubation period, the epidermal strips were peeled off from each section and photographed using a confocal microscope (Leica STELLARIS 8) to observe the internalization of the carbon dots. Imaging was conducted using excitation wavelength of 370 nm and emission wavelength of 450 nm. To assess the phytotoxicity effects, the samples were incubated in the dark for 48 h. Following the incubation period, the epidermal strips were peeled off from each section and stained with 5 mg/mL fluorescein diacetate and 2 mg/mL propidium iodide to distinguish live and dead cells, respectively. The samples were then incubated in the dark for 10 min. After incubation, confocal microscopy analysis was performed to evaluate the viability of epidermal cells.

### Infiltration of canola leaf tissues with CDs-RUBY

In this experiment, Canola seeds (*Brassica napus L*.) were surface sterilized using a 5% sodium hypochlorite solution for 10 min, followed by three washes with sterile distilled water. After sterilization, the seeds were sown in pots containing vermiculite to raise the seedlings. CDs and plasmid DNA containing the RUBY reporter gene were used to prepare CDs/plasmid DNA conjugates. The synthesized carbon dots and plasmid DNA were thoroughly mixed to form a conjugate with a final concentration of 30 ng/μL. After thorough vortexing, the mixture was incubated at 37°C for 30 min.

For the infiltration of the CDs/plasmid DNA into canola leaves, healthy leaves from 21 day-old canola seedlings were selected. Then, using a 1 mL needleless syringe, 100 µL of the CDs/plasmid DNA solution was carefully infiltrated into the leaves. The seedlings were then transferred to a growth chamber. After 7 days, the infiltrated leaves were visually inspected for phenotypic expression of the RUBY gene, followed by microscopic imaging to further validate the purple coloration, which indicates accumulation of betalains in the leaf cells (Jogam et al., 2024).

### Delivery of green fluorescent protein (sfGFP)-CDs complex into soybean roots and protoplast by passive diffusion mechanism

**FIGURE 2:**
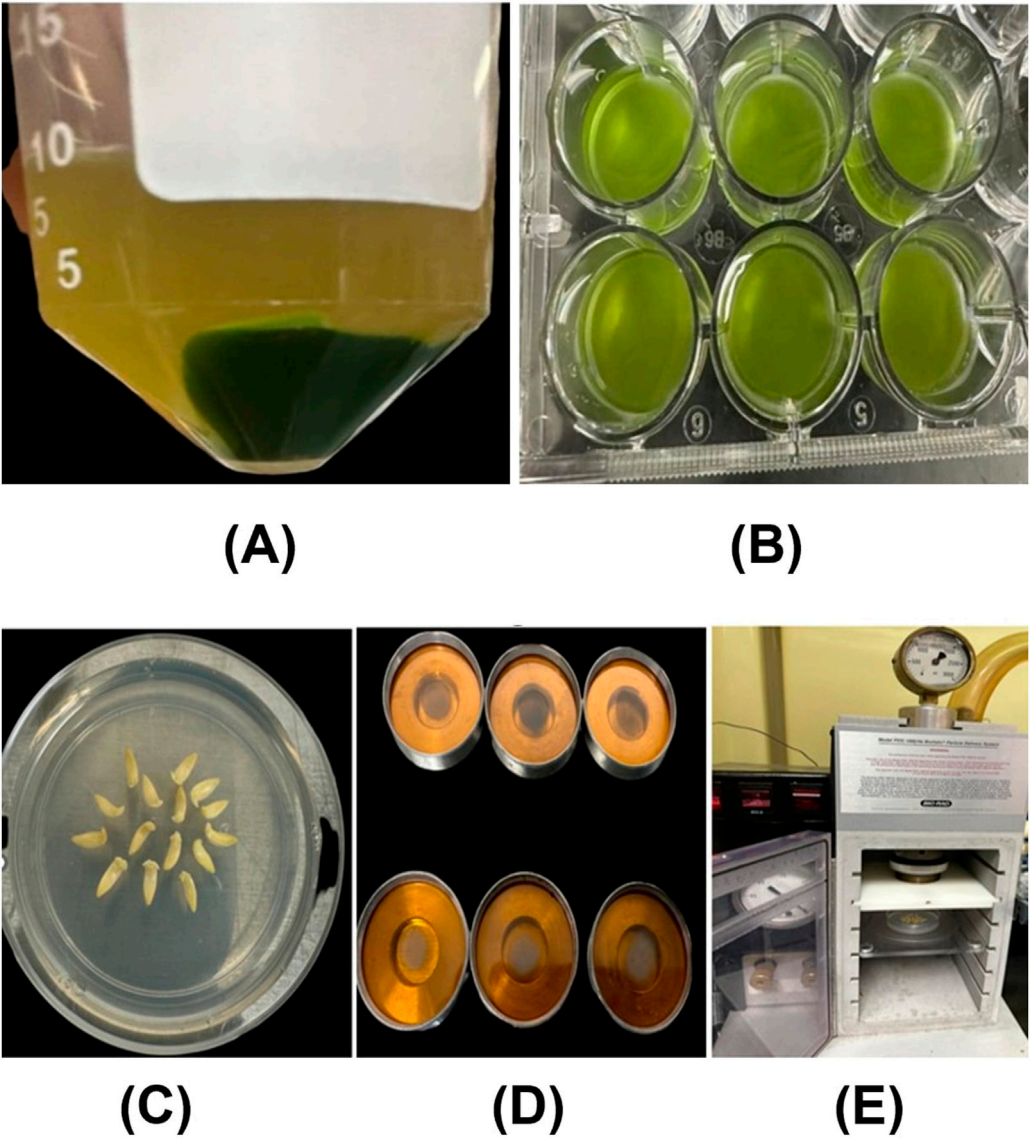
**(A)** Soybean protoplasts isolated through enzymatic digestion were resuspended in the W5 solution. **(B)** The isolated protoplasts were incubated in a CDs-sfGFP complex solution for 24 h **(C)** The embryonic axes of soybeans are arranged in a circular manner, with the apical meristematic region facing up in a petri dish containing bombardment media. **(D)** The carrier membranes contain tungsten-coated CD/Plasmid DNA (GUS reporter gene). **(E)** The soybean embryos, in a petri dish, positioned inside the gene gun for bombardment.

In this experiment, William 82 soybean seeds were initially surface sterilized using a solution of 30% bleach and 0.01% Triton-X for 15 min. Following sterilization, the seeds were washed three times with sterile distilled water. The sterilized seeds were then placed on germination paper to initiate germination. After 2 days, soybean cotyledons with root lengths of approximately 5 cm were selected and incubated at room temperature for 24 h in a premixed solution containing plasmid DNA with the sfGFP gene and CDs at a final concentration of 30 ng/μL. Post-incubation, the bottom portion of the cotyledons were photographed using confocal microscopy to evaluate the internalization of the CDs/pDNA complex and the expression of the GFP gene within the cells To deliver plasmid DNA-CDs complexes into soybean protoplasts, the following procedure was employed. Soybean protoplasts were isolated using the protocol reported by Wu and Hanzawa (2018), with minor modifications to suit our experimental needs. Leaves from 21 day-old soybean plants were collected, and small leaf strips (0.5–1 mm) were prepared from the middle portion of the leaves using a sterile razor blade, avoiding tissue wounding. The leaf strips were then submerged in an enzyme solution containing 1.5% cellulase (RPI, Cat# C32200, CAS No: 9012-54-8) and 0.4% macerozyme (RPI, Cat#M22010, CAS No: 9032-75-1) in a petri dish and subjected to vacuum infiltration for 30 min. After releasing the vacuum, the digestion continued at room temperature overnight. The solution was subsequently centrifuged at 80 RPM for 2 min to release the protoplasts. The mixture of enzymes and protoplasts was filtered through a 75 µm nylon mesh to remove undigested leaf material. To stop the digestion and enhance protoplast recovery, 2 mL of W5 solution (154 mM NaCl, 125 mM CaCl2, 5 mM KCl, 2 mM MES (pH 5.7) was added to the protoplast solution, followed by centrifugation at 1000 RPM for 2 min to obtain green pellets. These pellets were gently resuspended in W5 solution and kept on ice for 30 min. Afterward, the solution was centrifuged at 1000 RPM for 1 min, and the protoplasts were resuspended in MMG solution (0.4 M mannitol, 15 mM MgCl2, 4 mM MES (pH 5.7) (Figure 2A).

For the delivery of plasmid DNA containing the sfGFP gene and CDs into the protoplasts. Initially a CDs/plasmid DNA complex was prepared by gently mixing 5 µg of plasmid DNA with 100 µL (1.9 mg/mL) of carbon dots, followed by incubation at 37°C for 30 min. This complex was then slowly added, drop by drop, to 100 µL of protoplast solution and incubated in the dark at room temperature for 24 h (Figure 2B). After incubation, the solution containing the CDs/plasmid DNA complex and protoplasts was centrifuged at 600 RPM to pellet the protoplasts. The protoplasts were gently resuspended and subsequently photographed using confocal microscopy to assess the internalization of CDs and expression of the GFP gene. The mean fluorescence intensity in root cells and protoplast was analyzed using ImageJ software version 1.8.0. Statistical analysis using an independent two-sample *t*-test was performed with a custom Python script in Google Colaboratory (https://colab.research.google.com/). Statistically significant differences (P < 0.05) were denoted by distinct letters.

### Delivery of plasmids with different sizes into onion epidermal cells using GeneGun

To evaluate the ability of synthesized CDs to deliver plasmid DNA as a substitution for spermidine, we conducted an experiment using a gene gun to deliver plasmids into onion epidermal cells, with spermidine as a control, following the protocol by Li et al. (2000). Initially, 25 µL of 1 µM tungsten particles were vortexed and sonicated for 30 s. Then, 5 µg of plasmid DNA and 25 µL of 2.5 M CaCl₂ were added to the tungsten particles during sonication and mixed thoroughly. Subsequently, 10 µL of either spermidine (positive control) or CDs were added, and the mixture was homogenized by pipetting up and down while sonicating for 3 min. The solution was then centrifuged at 3000 RPM for 30 s. The supernatant was discarded, and the pellet was resuspended in 180 µL of absolute ethanol. The solution was centrifuged again at 3000 RPM for 30 s, the supernatant was removed, and the pellet was resuspended in 45 µL of absolute ethanol and stored at −20°C until bombardment.

For plasmid delivery into onion epidermal cells, fresh onions were cut into quarters, and sections of the epidermis (≥3 × 3 cm) were prepared and placed on water agar medium. The CDs/plasmid DNA coated tungsten particles were retrieved from −20°C, and 15 µL of the suspension was spread on the center of the carrier disk while sonicating. The carrier was then air-dried to facilitate the evaporation of ethanol. Bombardment was performed using a BioRad gene gun (Model PDS-1000/He Biolistic Particle Delivery System), following the protocol by Li et al. (2000).

After bombardment, the onion tissues were incubated in the dark at room temperature for 48 h. The epidermal strips were then peeled off using sterile forceps, laid on a slide, and the expression of reporter genes was examined using confocal microscopy and mean fluorescence intensity was analyzed using ImageJ software version 1.8.0. Statistical analysis, including ANOVA and Tukey’s test, was conducted using a custom Python script in Google Colaboratory (https://colab.research.google.com/). Categories with statistically significant differences (P < 0.05) were indicated with distinct letters.

### CDs-plasmid DNA delivery into soybean embryos using biolistic method

In this experiment, Williams 82 soybean seeds were surface sterilized using 30% bleach and 0.01% Triton-X for 15 min, followed by three washes with sterile distilled water. After sterilization, the seeds were incubated in sterile distilled water at room temperature overnight in a conical flask. Following incubation, the soybean embryos were separated from the cotyledons using sterile micro forceps and a stainless-steel blade and transferred to bombardment media consisting of Murashige and Skoog salt, 3% sucrose, and 0.5% agar, adjusted to a final pH of 5.7 (Figure 2C).

The preparation of CDs/plasmid DNA-coated tungsten particles and bombardment was performed similar to the previous experiment (Li et al., 2000) (Figures 2D, E). Immediately after bombardment, the embryos were transferred to shoot induction media containing Murashige and Skoog salt, 22.2 mM BAP, 3% sucrose, and 0.5% agar, with a final pH of 5.7, and incubated in the dark for 24 h. Subsequently, the embryos were transferred to the culture media containing Murashige and Skoog salt, 3% sucrose, and agar, supplemented with kanamycin and 5 mg/L silver nitrate. After 7 days, the embryos were removed from the media, and a GUS gene expression assay was performed using the protocol established by Jefferson (1987). The embryos were then kept in a 95% ethanol solution overnight to remove chlorophyll, followed by transient GUS expression analysis using a stereo microscope.

## Results and discussion

CDs were synthesized in a one-pot method using microwave-assisted synthesis, with citric acid as the carbon source and β-alanine, an amino acid derivative, as the passivating agent to minimize phytotoxicity. The pH of the buffer used during the synthesis played a critical role in determining the effectiveness of gene delivery. CDs synthesized at lower pH levels (pH 3–5) demonstrated superior gene delivery efficacy compared to those synthesized at higher pH levels (Pandey et al., 2021). This enhanced efficacy can be attributed to differences in the size and density of oxygen-containing functional groups, such as hydroxyl (-OH), carboxyl (-COOH), and carbonyl (C=O), on the surface of the CDs (Wongso et al., 2019). At lower pH, the synthesis environment promotes the formation of a higher density of these functional groups, which catalyze the carbonization process by effectively fragmenting larger precursor molecules into smaller clusters (Ehtesabi et al., 2020). This prevents the formation of larger carbonaceous structures, resulting in smaller CDs. Additionally, the abundant functional groups improve the solubility and stabilization of the smaller CDs in the synthesis medium, preventing aggregation of CDs and maintaining their actual size (Mansuriya and Altintas, 2021), which is critical for efficient conjugation with plasmid DNA.

Smaller CDs possess better penetration capabilities in comparison to their larger counterparts synthesized at higher pH levels (Jung et al., 2015; Wongso et al., 2019; Zhang et al., 2020). Furthermore, synthesizing CDs at lower pH levels facilitates the protonation of hydroxyl groups (-OH) on their surface, reducing the negative charge. This reduction in surface charge minimizes the electrostatic repulsion between the negatively charged plasmid DNA and the CDs, thereby increasing the binding efficiency of the plasmid DNA with the carbon dots.

**FIGURE 3:**
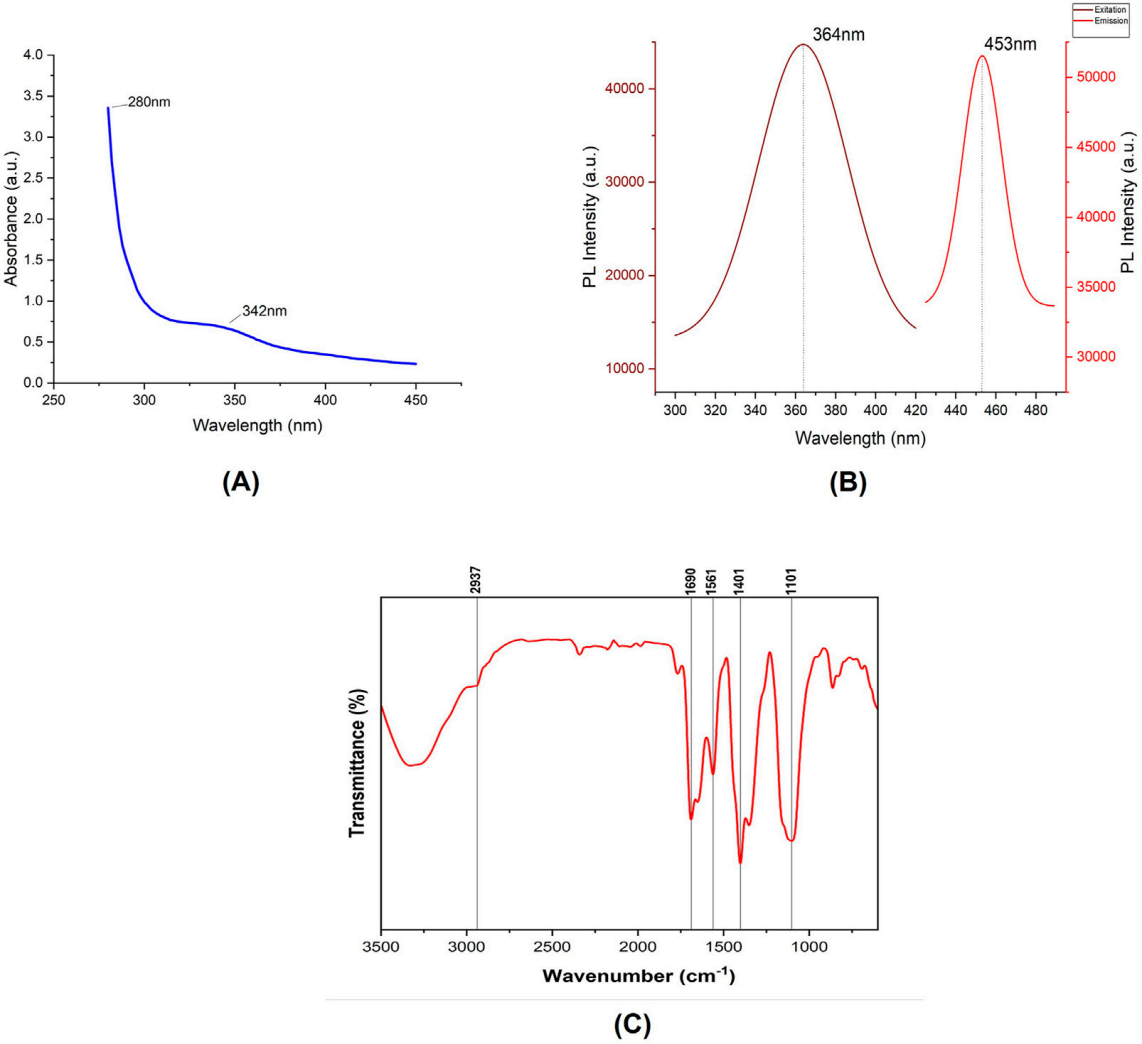
**(A)** Absorption spectrum of the synthesized CDs, displaying prominent peaks at 280 nm and 342 nm. **(B)** Fluorescence spectra of the synthesized CDs, including excitation and emission spectra. **(C)** FTIR spectrum illustrating the functional groups present in the Citric acid/β-alanine-derived carbon dots.

**FIGURE 4:**
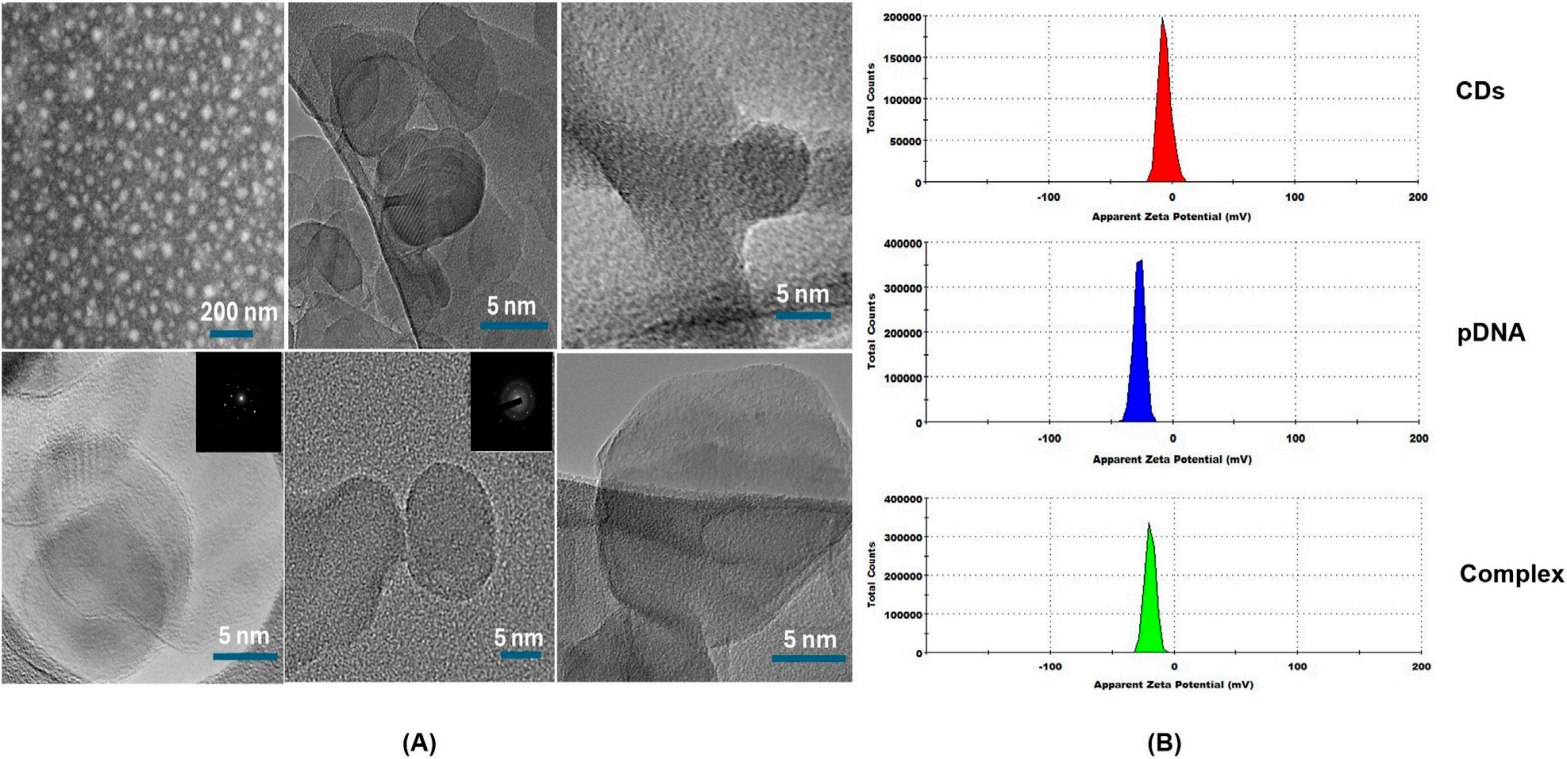
**(A)** High-resolution transmission electron microscopy (HRTEM) and Dark-field scanning transmission electron microscopy (STEM) images of carbon dots. **(B)** Zeta-potential of carbon dots (CDs), plasmid DNA (pDNA) and the complex of carbon dots and plasmid DNA (Complex).

Following the synthesis of carbon dots, their characterization was carried out to investigate their photoluminescent properties. The analysis revealed two distinct absorption peaks at 280 nm and 342 nm (Figure 3A) (Kim et al., 2014; Choi et al., 2014). The peak observed at 280 nm is indicative of the presence of an aromatic system within the carbon dots, signifying the existence of an sp^2^ carbon network. Meanwhile, the peak at 342 nm corresponds to the n–π* electronic transition of carbonyl functional groups presents on the surface of the synthesized carbon dots (Mewada et al., 2014). The fluorescence characterization of the carbon dots revealed a maximum emission wavelength at 453 nm when excited at 364 nm, which aligns with common characteristics reported for carbon dots in previous studies (Figure 3B) (Carbonaro et al., 2018). Additionally, quantum yield analysis was conducted using Rhodamine B as a reference. The results of this analysis determined the quantum yield of carbon dots to be 18.75% (Pandiyan et al., 2020). The FTIR spectra of the synthesized carbon dots were obtained from a liquid sample to analyze the surface characteristics and functional groups present in the material (Figure 3C). The FTIR analysis revealed several significant peaks. A prominent peak at 2,937 cm⁻^1^ was assigned to C-H stretching vibrations, characteristic of the alkane group. The peak at 1,401 cm⁻^1^, corresponding to C-N stretching, is particularly notable as it indicates the successful passivation of the carbon dots with beta-alanine. Furthermore, a peak at 1,690 cm⁻^1^, attributed to C=O stretching, suggests the presence of ketone groups. Another peak at 1,101 cm⁻^1^ was identified, corresponding to C-O stretching vibrations related to the stereochemistry of the carbonyl group. These findings collectively confirm the successful synthesis and passivation of carbon dots with beta-alanine (Pandey et al., 2021; Jung et al., 2015). Recent studies have shown that nanoparticles with diameters up to 50 nm are capable of permeating plant cell walls, although the exact mechanisms underlying this process remain unclear (Wang et al., 2016; Schwab et al., 2016). Furthermore, research has suggested that nanomaterials with sizes around 20 nm can be successfully internalized in plant species such as tobacco, wheat, and cotton (Demirer et al., 2019a; Giraldo et al., 2014). To evaluate the particle size of the synthesized carbon dots, which is a critical parameter for their application in gene delivery, STEM and TEM imaging were performed. The analysis revealed a particle size distribution ranging from approximately 5 nm–20.2 nm, with an average size of 13.5 ± 5.6 nm, based on measurements of 266 individual particles, which is similar to the carbon dots reported by (Meloni et al., 2022; Zhang et al., 2018; Pandiyan et al., 2020; Pan et al., 2020). These findings indicate that carbon dots with this size range are likely capable of bypassing plant cell wall barriers. Most of the carbon dots exhibited a spherical morphology, confirming the successful synthesis of zero-dimensional (0D) structures. However, occasional observations of layered carbon structures resembling two-dimensional (2D) graphene sheets were also noted (Figure 4A). Selected Area Electron Diffraction (SAED) patterns revealed bright spots, indicating the crystalline nature of the synthesized particles. The SAED patterns displayed distinct hexagonal symmetry, characterized by bright diffraction spots and rings. These features are hallmark indicators of graphitic materials, reflecting the crystalline arrangement of sp^2^-hybridized carbon atoms. The observed hexagonal symmetry strongly indicate that the primary structural component of the synthesized carbon dots is sp^2^-hybridized carbon, consistent with the typical properties of graphitic domains.

**FIGURE 5:**
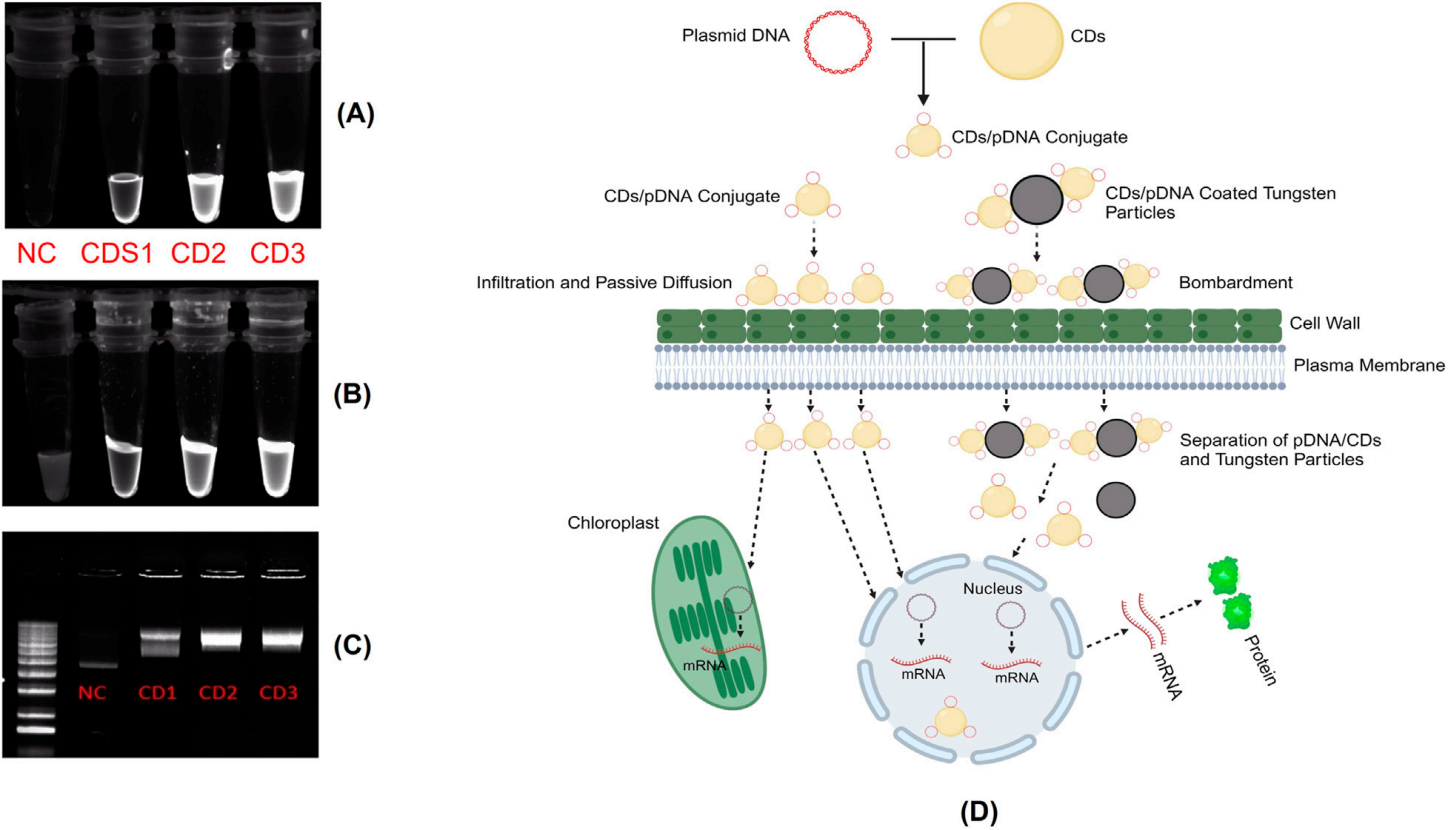
**(A)** Assessment of the fluorescent properties of CDs compared to plasmid DNA without loading dye. **(B)** Assessment of the fluorescent properties of CDs compared to plasmid DNA with loading dye. **(C)** Binding analysis of Carbon Dots (CDs) to plasmid DNA. Agarose gel electrophoresis showing naked plasmid DNA (Lane 1) and CDs/DNA complexes at different concentrations of CDs: 4.75 μg/μL (CD1), 6.3 μg/μL (CD2), and 9.5 μg/μL (CD3) in Lanes 2 to 4, respectively. **(D)** Mechanism of internalization of the CDs/plasmid DNA complex into plant cells and the subsequent transient gene expression involves several steps. Upon application of the CDs/plasmid DNA complex through various insertion techniques such as infiltration, passive diffusion, or particle bombardment. The CDs/plasmid DNA complex passes through the plant cell wall and membrane to ultimately reach the cytosol. In the cytosol, the plasmid DNA could either detach and enter the chloroplast and nucleus independently or be transported by the CDs across the membranes to initiate gene expression.

To evaluate the surface electric charge of the synthesized CDs, zeta potential analysis was performed. The CDs exhibited a zeta potential of −5.76 ± 4.14 mV, indicating a slightly negative surface charge. This negative charge is attributed to the presence of two negatively charged functional groups, specifically C=O and C-O, on the surface. Additionally, this negative surface charge contributes to the colloidal stability of the CDs. To assess the ability of the CDs to conjugate with plasmid DNA, a critical step in the gene delivery process, the plasmid DNA was mixed with the CDs and incubated at 37°C for 30 min. Zeta potential analysis of the mixture, containing plasmid DNA with an initial zeta potential of −27 ± 4.3 mV and CDs, revealed a significant decrease in the zeta potential to −19.2 ± 4.14 mV (Figure 4B). This change confirms the successful interaction between the negatively charged phosphate backbone of the plasmid DNA and the surface of the CDs, which contains positively charged amine groups introduced during the passivation process with β-alanine. This interaction results in the amine groups neutralizing part of the negatively charged DNA phosphate groups, leading to the observed reduction in zeta potential. The decrease in zeta potential provides strong evidence of effective electrostatic interaction and conjugation between the negatively charged plasmid DNA and the surface of the CDs, which is crucial for their potential application in gene delivery systems. These findings align with studies showing that negatively charged nanoparticles, such as nanohydroxyapatites functionalized with arginine, can successfully bind plasmid DNA and achieve gene delivery due to amine groups introduced by arginine (Izuegbunam et al., 2021). Similarly, negatively charged carbon dots have been shown to bind plasmid DNA and deliver genes effectively, facilitated by amine functionalization (Pandey et al., 2021). Recent studies have shown that negatively charged CDs can move through plant roots using both symplastic and apoplastic pathways, whereas positively charged CDs mainly use the apoplastic pathway. Negatively charged CDs were also found to move more efficiently from roots to leaves compared to positively charged ones. This indicates that a negative charge can improve the delivery efficiency of CDs in plants. As a result, negatively charged CDs complexed with plasmid DNA, with overall negative charge, offer a promising approach for effective gene delivery into plant tissues (Chen et al., 2024). To further assess the capacity of the synthesized CDs to load plasmid DNA, a binding efficiency investigation was conducted using various concentrations of CDs. CDs at concentrations of 9.5 μg/μL, 6.3 μg/μL, and 4.75 μg/μL were mixed with plasmid DNA and incubated for 30 min at 37°C. The CDs-plasmid DNA complexes and a negative control (naked DNA) were then subjected to agarose gel electrophoresis at 85 V for 60 min, and the results were visualized using ultraviolet irradiation. The delay in the movement of the complex compared to plasmid DNA on the gel was attributed to its lower negative charge and higher molecular weight, as the complex consists of both plasmid DNA and carbon dots. This also resulted in the appearance of larger bands in the lanes containing the CDs-plasmid DNA mixture. The darker bands observed in the assay compared to the negative control were attributed to the fluorescent properties of the CDs. This was confirmed by exposing the complex mixtures and naked pDNA to UV light without adding loading dye, where significant fluorescence was observed in the complex mixtures, but not in the pDNA, confirming the fluorescence property of the CDs (Figure 5A). After adding loading dye, fluorescence was observed in all the tubes. However, the complex showed significantly higher intensity due to the combined fluorescence properties of the CDs and the loading dye, whereas the fluorescence in pDNA was solely due to the loading dye (Figure 5B). Hence, the delay in the movement of the complex and the appearance of larger and darker bands compared to plasmid DNA confirm the efficient binding of plasmid DNA with CDs, aligning with the findings of the zeta potential studies (Figure 5C). Our hypothesis for this study was that the positively charged amine groups on the surface of CDs would electrostatically interact with the negatively charged plasmid DNA, thereby facilitating its passage through the plant cell wall and membrane to ultimately reach the cytosol. In the cytosol, the plasmid DNA could either detach and enter the nucleus and chloroplast independently or be transported by the CDs across the membranes to initiate gene expression (Figure 5D).

**FIGURE 6:**
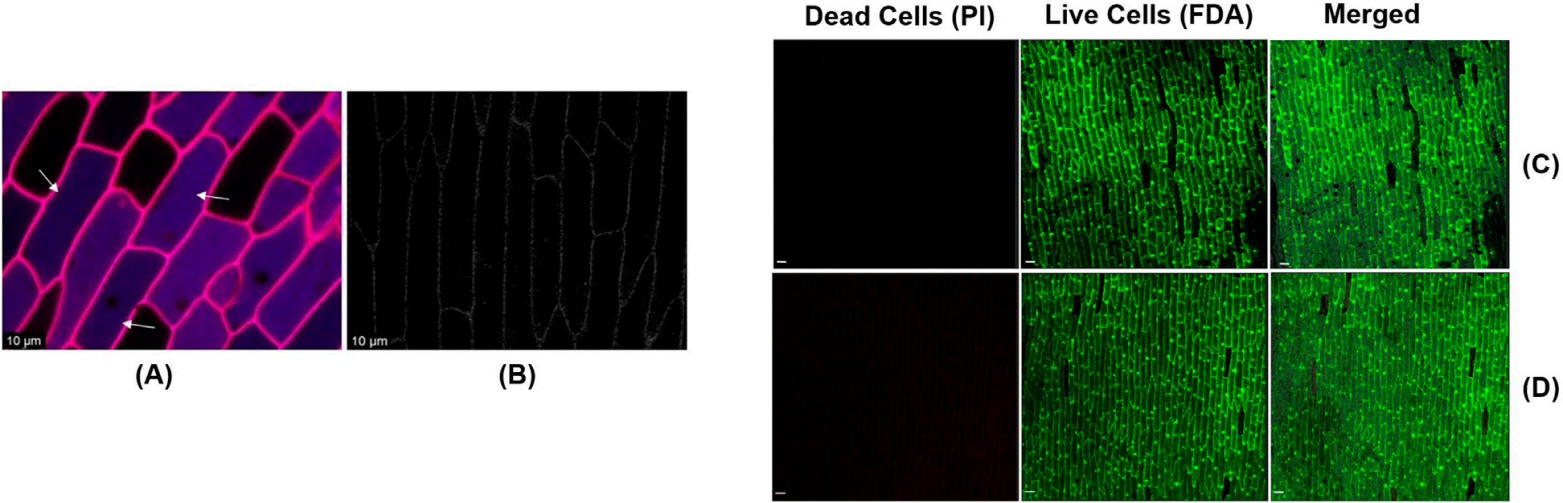
**(A)** The confirmation of internalization of CDs (Indicated by the arrows) into onion epidermal cells was achieved through confocal imaging the epidermal cells after they were sprayed with CDs and incubated at room temperature for 20 min (scale bar = 10 μm). **(B)** Absence of blue fluorescent signals in onion epidermal cells sprayed with sterile water and incubated at room temperature for 20 min (Control) (scale bar = 10 μm). Assessment of the phytotoxic effects of carbon dots on onion epidermal cells using PI and FDA staining. **(C)** Confocal image of the epidermal cells after treatment with carbon dots and incubation at room temperature for 48 h (scale bar = 100 μm). **(D)** Confocal image of the epidermal cells after treatment with sterile water and incubation at room temperature for 48 h (scale bar = 100 μm).

To investigate whether the synthesized CDs can internalize into plant tissues, we applied 10 µL of CDs onto sections of onion epidermis. After a 20 min incubation at room temperature, we conducted a confocal microscopy analysis using excitation wavelength of 370 nm and emission wavelength 450 nm to confirm the internalization of CDs into the epidermal cells. The appearance of blue, fluorescent signals during confocal microscopy revealed significant internalization of the CDs into the onion epidermal cells within the 20 min incubation period (Figure 6A), whereas the signal was absent under the control condition (Figure 6B). To assess the phytotoxicity of the synthesized carbon dots, onion epidermal samples were treated with carbon dots at a concentration of 19 mg/mL, consistent with the concentration used for internalization studies. The samples were incubated in the dark for 48 h. Following incubation, the cells were stained with propidium iodide (PI) and fluorescein diacetate (FDA) to evaluate cell viability. PI selectively binds to the DNA of dead cells, emitting red fluorescence, while FDA is enzymatically converted into the green fluorescent compound fluorescein by the esterase activity of viable cells, indicating cell viability (Jones et al., 2016). Confocal microscopy showed that most onion epidermal cells remained viable, with no signs of cell death after treatment (Figure 6C). These results were similar to the control group treated with sterile water, indicating that the carbon dots had no toxic effects on the cells (Figure 6D). These findings are consistent with previous reports indicating that carbon dots do not cause adverse effects on plant cells (Wang et al., 2020). Onion epidermal cells, known for their high sensitivity, exhibited no toxic effects when exposed to the carbon dots. In comparison, other plant tissues, being more structurally robust, are not expected to exhibit toxicity. These findings indicate that carbon dots are safe and suitable for use as carriers in gene delivery applications for plant tissues. To ensure that the synthesized CDs can act as selective plasmid DNA carriers in intact canola leaves, leading to transient expression of the RUBY reporter gene, we mixed the CDs with plasmid DNA containing the RUBY reporter gene at a final concentration of 30 ng/μL. This mixture was incubated for 30 min at 37°C to form a complex. The CDs-plasmid DNA complex was then infiltrated into 21 day-old canola leaves using a needleless syringe.

**FIGURE 7:**
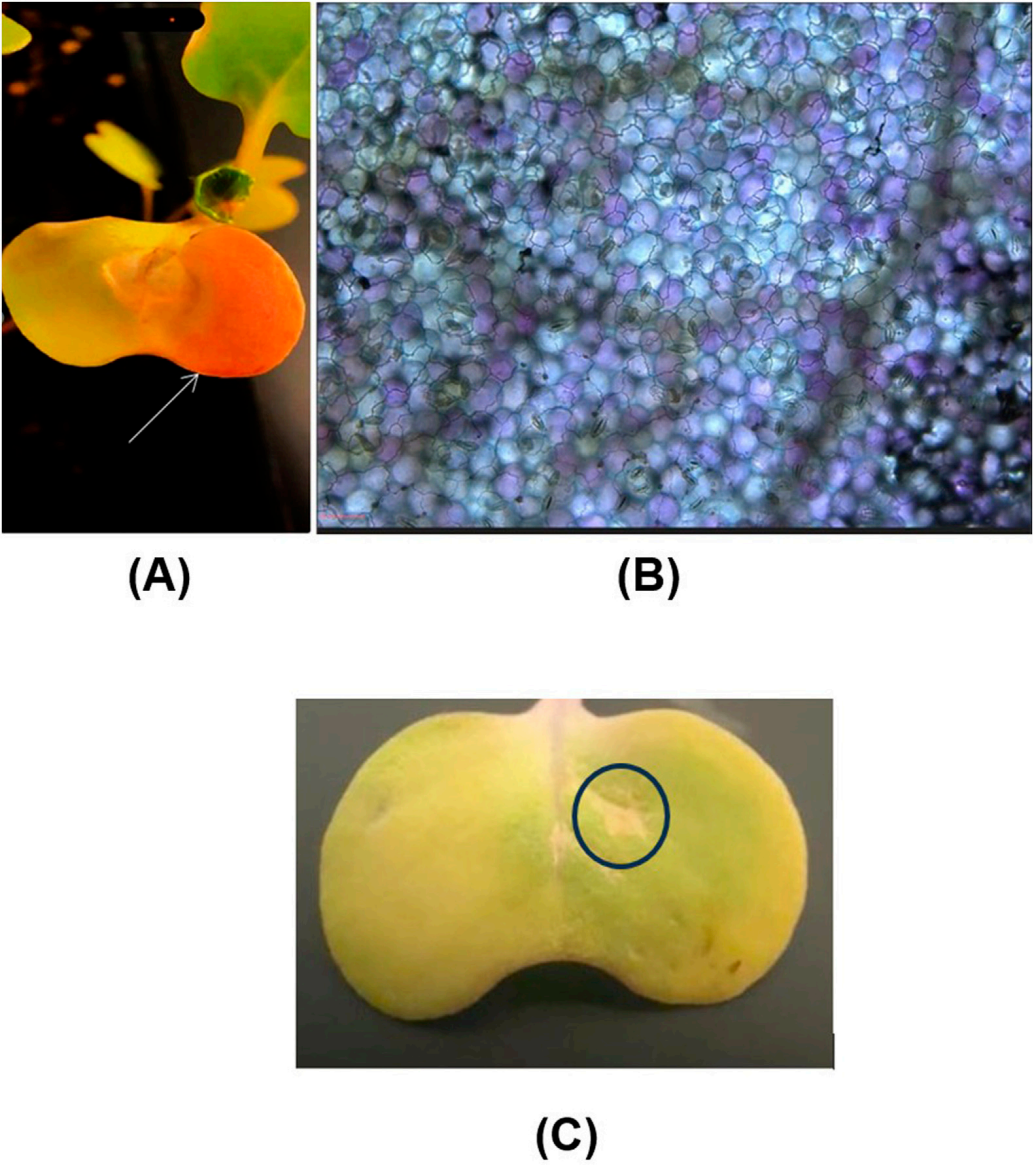
Delivery of CDs/plasmid DNA containing the RUBY reporter gene into intact canola leaves through infiltration. **(A)** Intact canola leaves were infiltrated with a 30 ng/μL CDs/plasmid DNA (RUBY) complex using a needleless syringe. The phenotypic expression of the RUBY gene (indicated by the white arrow) is visible in the leaf after 7 days of infiltration. **(B)** The expression of the ruby red gene in canola leaf cells was further confirmed through microscopy analysis (scale bar = 25 μm). **(C)** The absence of RUBY gene expression in canola leaf which is injected with plasmid DNA alone.

Following the infiltration, the leaves exhibited a clearly visible slight pinkish coloration after 7 days, indicating the expression of the RUBY gene in the infiltrated intact canola leaves (Figure 7A), whereas the same coloration was not noticed in the case of control condition where the leaf was injected with plasmid DNA alone (Figure 7C). To further confirm the expression of the RUBY gene within the cells, microscopy analysis was performed, revealing a vibrant purple coloration in the epidermis, consistent with previous findings (Jogam et al., 2024). This observation confirms the accumulation of betalain in the epidermis. Notably, betalain accumulation was detected in approximately 34.5% of the epidermal cells, indicating partial but significant gene expression within the tissue. (Figure 7B). This investigation demonstrates the effectiveness of our carbon dots in delivering plasmid DNA into leaf tissues via the infiltration technique, thereby facilitating functional DNA insertion. Additionally, it suggests that these carbon dots can be employed in other plant species for delivering functional DNA using the same infiltration technique. Syringe infiltration is a versatile technique widely used in plant genetic engineering. It facilitates the rapid assessment of transient gene expression, evaluation of gene knockdown efficiency using siRNA and dsRNA either alone or in combination with nanocarriers and gene knockout using CRISPR technologies (Přibylová et al., 2022). The use of siRNA or dsRNA for knocking down genes of interest in disease and pest management has been shown to be significantly more efficient when applied using nanoparticles as carriers, as they increase stability compared to their naked application. This makes syringe infiltration a valuable tool, regardless of the type of carrier employed (Lei et al., 2020). Hence, the synthesized CDs can be used in such applications to further advance plant protection and plant genetic engineering.

**FIGURE 8:**
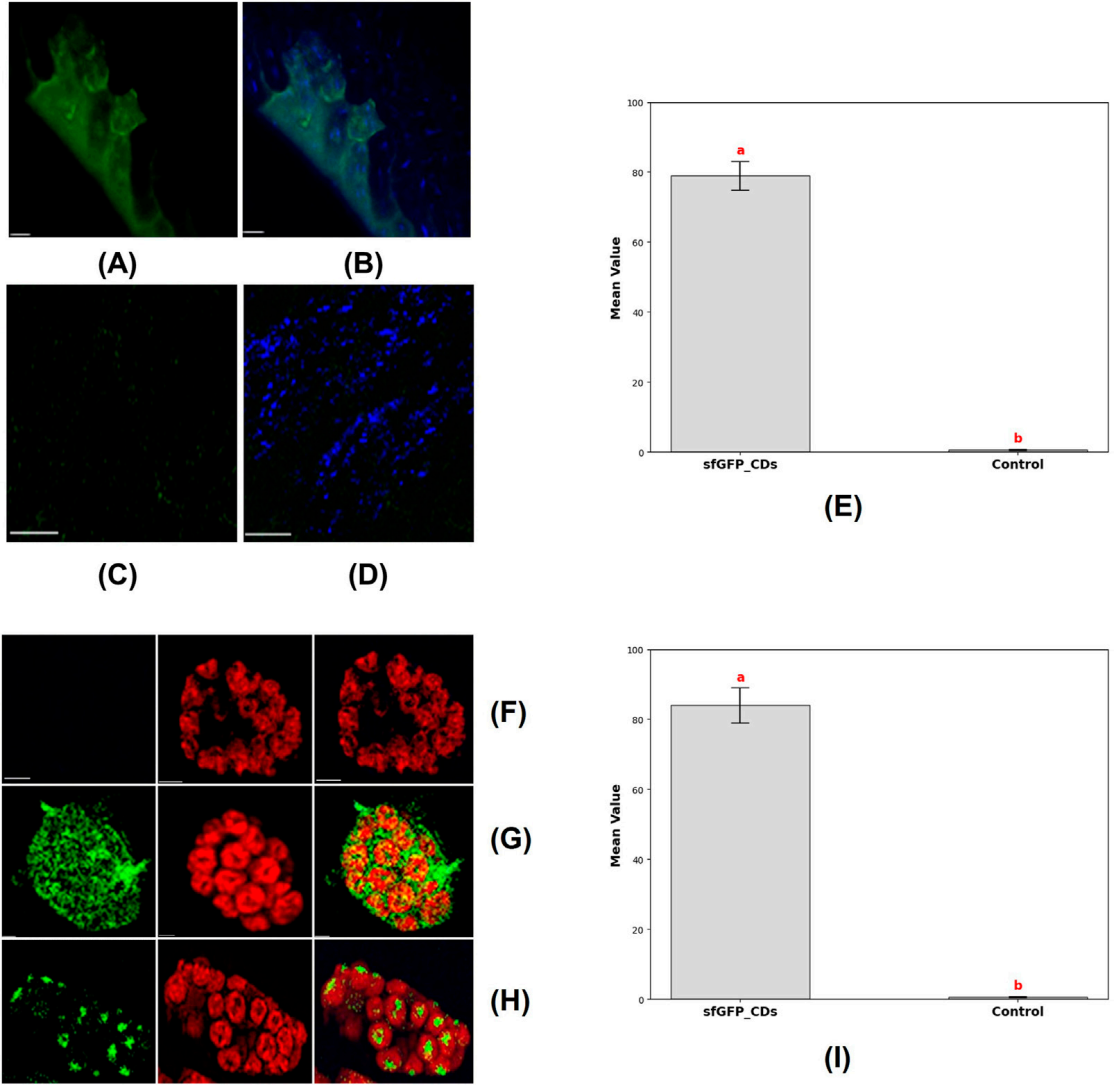
Delivery of CDs-sfGFP into soybean root cells and protoplast was achieved by incubating them in a CDs-sfGFP complex for 24 h and subsequent expression analysis via confocal imaging. **(A)** Transient GFP reporter gene expression was observed in the soybean root cells after incubation in a 30 ng/μL CDs-sfGFP complex solution for 24 h (scale bar = 25 μm). **(B)** The merged image shows GFP expression and the internalization of carbon dots into root cell (scale bar = 25 μm). **(C)** Absence of transient GFP reporter gene expression in the soybean root cells after incubation in a solution containing plasmid DNA alone for 24 h (scale bar = 25 μm). **(D)** The internalization of carbon dots into root cell after incubation in a CDs solution alone for 24 h (scale bar = 25 μm). **(E)** Graph representing the mean fluorescence fntensity of GFP in root cells, where the plasmid was delivered using CDs as carrier (sfGFP_CDs) and without CDs as a carrier (Control). Different letters indicate significant differences between treatments (*P* < 0.0001). **(F)** Absence of transient expression of GFP fusion proteins in isolated soybean protoplasts and chloroplast, after incubation in the solution containing plasmid DNA alone (Scale bar = 5 μm). **(G)** Transient expression of GFP fusion proteins in isolated soybean protoplasts (Scale bar = 5 μm) **(H)** Transient expression of GFP fusion protein in chloroplast, images showing GFP and chlorophyll autofluorescence, indicative of fluorescent expression, along with merged and bright field images, are provided (Scale bar = 5 μm). **(I)** Graph representing the mean fluorescence intensity of GFP in protoplast, where the plasmid was delivered using CDs as a carrier (sfGFP_CDs) and without CDs as a carrier (Control). Different letters indicate significant differences between treatments (*P* < 0.0001).

After that, we investigated the potential of synthesized CDs as carriers for functional DNA into root cells. Plant cell walls are traditionally regarded as barriers to biomolecule transport, with a reported size exclusion limit of approximately 5–20 nm (Cunningham et al., 2018). Despite this, recent studies have demonstrated that nanoparticles with diameters of up to 50 nm can penetrate cell walls, though the mechanisms underlying this process remain unclear (Wang et al., 2016; Schwab et al., 2016). Notably, nanoparticles with sizes around 20 nm have been successfully internalized in tobacco, wheat, and cotton plants (Demirer et al., 2019a). Furthermore, negatively charged carbon dots have shown superior effectiveness in reaching the roots of cucumber and cotton plants compared to positively charged CDs. Additionally, negatively charged CDs exhibit more efficient translocation from roots to leaves in cucumber and cotton, highlighting their potential for enhanced delivery efficiency in plants. We hypothesized that CDs with a diameter of 13.5 nm could bypass this barrier and facilitate the internalization of plasmid DNA containing the sfGFP reporter gene into root cells without the need for external agents like gene guns or ultrasound. Soybean seeds were germinated on germination paper until they reached a root length of 3–4 cm. To prepare the DNA delivery system, CDs were mixed with plasmid DNA containing the GFP reporter gene to a final concentration of 30 ng/μL and incubated at 37°C for 30 min. The soybean cotyledons with roots were then incubated in the CDs/plasmid DNA conjugate solution for 24 h. Confocal microscopy analysis of the demonstrated the expression of transient GFP inside the root cells and cytoplasm (Figures 8A,B), suggesting that our CDs successfully delivered the plasmid DNA into root cells and facilitated subsequent gene expression via a cellular uptake mechanism, whereas the absence of GFP gene expression was observed in the control conditions (Figures 8C, D). The expression of the reporter gene was quantified by measuring mean fluorescence intensity resulting from the delivery of the plasmid using CDs as a carrier compared to plasmid delivery without a carrier. The results showed a mean fluorescence intensity of GFP in root cells of 78.92 ± 4.17, while GFP expression was completely absent under the control condition (Figure 8E). This study highlights the effectiveness of CDs as carriers for DNA delivery into plant root cells. Hence, these carbon dots can be utilized for the delivery of various gene-editing constructs, such as siRNA, dsRNA, and CRISPR constructs, through passive diffusion mechanisms via root feeding. The negatively charged carbon dots have demonstrated the capability to efficiently translocate from the roots to various parts of the plant (Chen et al., 2024). Therefore, the synthesized carbon dots present a promising option as carriers for gene knockout or knockdown applications, targeting genes with significant expression in different parts of the plant through root feeding.

In this next investigation, we aimed to demonstrate the delivery of DNA into protoplast and chloroplasts, subsequent transient expression of reporter genes using manufactured CDs as carriers for plasmid DNA. We obtained soybean mesophyll protoplasts from 21 day-old soybean plants through an enzymatic digestion technique. The CDs were mixed with plasmid DNA containing the GFP reporter gene and incubated at 37°C for 30 min to facilitate binding. Subsequently, the protoplasts were cultured with the CDs-plasmid DNA complex for 24 h in a dark environment at room temperature. Confocal microscopy analysis following the incubation period showed that the CDs-plasmid DNA conjugate had penetrated the protoplasts and chloroplast membranes, localizing within the chloroplasts (Figure 8G). This was confirmed by monitoring the autofluorescence of chlorophyll and the combined image of GFP expression and chlorophyll autofluorescence, which exhibited the complete co-localization of GFP and the chloroplasts (Figure 8H). Absence of transient expression of GFP fusion proteins was noticed in the isolated soybean protoplasts and chloroplasts following incubation with the solution containing plasmid DNA alone (Figure 8F). The expression of the reporter gene was quantified by measuring mean fluorescence intensity following plasmid delivery with CDs as a carrier and without a carrier. In protoplasts, the mean fluorescence intensity of GFP was 83.9 ± 5.03, whereas no GFP expression was detected in the control condition where no carrier was used (Figure 8I). Therefore, our CDs-mediated functional DNA delivery system allows for the quick and passive diffusion of plasmid DNA into protoplasts, resulting in efficient transgenic expression without any noticeable negative impact on protoplast viability. This study demonstrates the potential of CDs as effective carriers for functional DNA delivery into chloroplasts.

**FIGURE 9:**
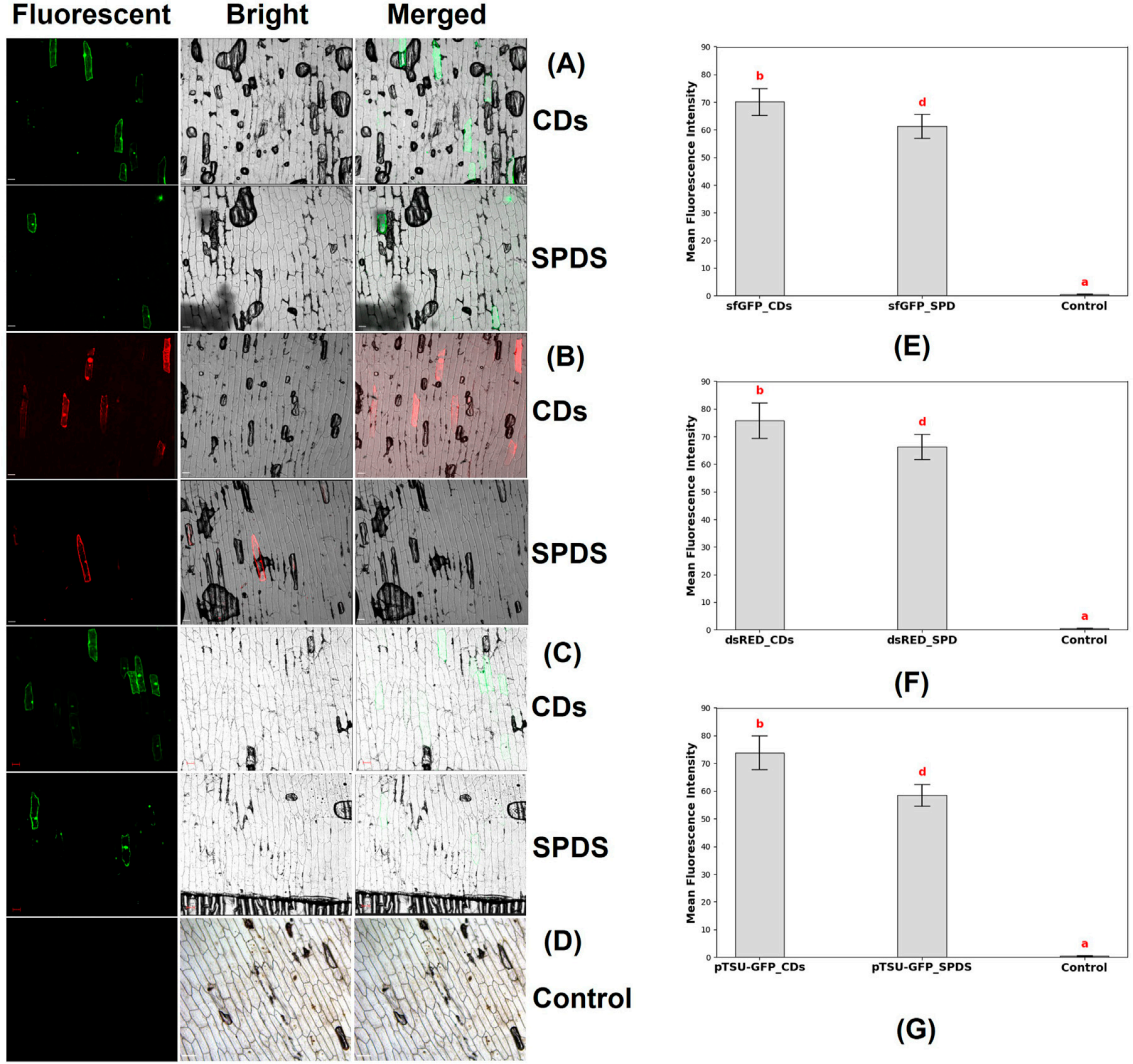
Confocal microscopy images of the epidermal cells of onion, which were bombarded with CDs/plasmid DNA coated microparticles and spermidine/plasmid DNA coated microparticles. **(A)** Fluorescence field, bright field, and merged field images of onion epidermal cells 48 h after bombardment with CDs/plasmid DNA-coated microparticles and spermidine (SPDS)/plasmid DNA-coated microparticles. The expression of the sfGFP reporter gene (4 kb) was observed (scale bar = 100 μm). **(B)** Fluorescence field, bright field, and merged field images of onion epidermal cells 48 h after bombardment with CDs and spermidine as DNA carriers, showing the expression of the dsRED reporter gene (11 kb) in the onion epidermal cells (scale bar = 100 μm). **(C)** Fluorescence field, bright field, and merged field images of onion epidermal cells 48 h after bombardment with CDs and spermidine as DNA carriers, showing the expression of the CRISPR construct (17 kb) in the onion epidermal cells (scale bar = 100 μm). **(D)** Absence of fluorescent signals in onion epidermal cells where neither spermidine nor carbon dots were used as binding agents to coat the DNA with tungsten particles before bombardment into the epidermal cells (Control). **(E)** Graph representing the mean fluorescence intensity of sfGFP reporter gene in epidermal cells, where the plasmid was delivered using CDs (sfGFP_CDs), spermidine (sfGFP_SPD) as binding agents, and without a binding agent (control). Different letters indicate significant differences between treatments (P < 0.0001). **(F)** Graph representing the mean fluorescence intensity of dsRED reporter gene in epidermal cells, where the plasmid was delivered using CDs (dsRED_CDs), spermidine (dsRED _SPD) as binding agents, and without a binding agent (control). Different letters indicate significant differences between treatments (P < 0.0001). **(G)** Graph representing the mean fluorescence intensity of pTSU-GFP reporter gene in epidermal cells, where the plasmid was delivered using CDs (pTSU-GFP _CDs), spermidine (pTSU-GFP _SPD) as binding agents, and without a binding agent (control). Different letters indicate significant differences between treatments (P < 0.0001).

To explore the potential of carbon dots as a substitute for spermidine in plant genetic engineering, we investigated their ability to facilitate the binding of negatively charged DNA with negatively charged tungsten microcarriers. Furthermore, we aimed to determine the maximum plasmid size that could be introduced into plant tissues using these CDs. This study focused on testing plasmids of varying sizes, including 35Sp_sfGFP_nosT (4 kb), pPSU1-dsRED (11 kb), and a CRISPR construct, pTSU-GFP (17 kb), to evaluate the versatility and efficiency of CDs in DNA delivery.

The experiment began with the preparation of onion epidermal sections measuring ≥3 × 3 cm, which were placed on water agar medium. Tungsten microparticles were coated with plasmid DNA and CDs, or alternatively with plasmid DNA and spermidine, to serve as the delivery vehicles. These prepared microcarriers were then bombarded into the onion epidermal cells. Following the bombardment, the samples were incubated in the dark at room temperature for 48 h. After incubation, confocal microscopy was used to analyze the expression of the plasmids in the epidermal cells. Microscopy analysis revealed the expression of reporter genes, such as sfGFP in both CDs and spermidine (Figure 9A), DsRed in CDs and spermidine (Figure 9B), and pTSU-GFP in both CDs and spermidine (Figure 9C). And the absence of gene expression was observed in the control group, where neither carbon dots nor spermidine were used as binding agents between the DNA and tungsten particles (Figure 9D). The expression of the reporter gene was quantified by measuring fluorescence resulting from plasmid delivery using CDs and spermidine as binding agents, compared to delivery without a binding agent. For the smallest plasmid, 35Sp_sfGFP_nosT (4 kb), the mean fluorescence intensity was significantly higher with CDs (70.15 ± 4.86) compared to spermidine (61.2 ± 4.32) (Figure 9E). Similarly, for the medium-sized plasmid, pPSU1-dsRED (11 kb), CDs achieved a mean fluorescence intensity of 75.8 ± 6.44, exceeding that of spermidine (66.3 ± 4.5) (Figure 9F). Notably, the largest plasmid, pTSU-GFP (17 kb), also demonstrated enhanced expression with CDs (73.85 ± 6.06) compared to spermidine (58.4 ± 3.9) (Figure 9G). Overall, this study demonstrates that carbon dots are a highly effective alternative to spermidine for DNA delivery in plant tissues. However, this study focuses solely on plasmid DNA delivery and does not evaluate gene editing or gene knockdown efficiency. While CDs have shown significant promise in facilitating DNA binding and delivery with higher fluorescence intensity compared to spermidine, these findings only confirm their potential as an alternative for plasmid delivery, not their overall efficiency in other applications. To establish their efficacy as a reliable replacement for spermidine, further research is necessary to assess their performance in gene editing and gene knockdown techniques. In conclusion, this study demonstrates that CDs synthesized using citric acid and β-alanine are a highly effective alternative to spermidine for DNA delivery in plant tissues. The results indicate that these CDs can effectively deliver plasmids up to 17 kb, including CRISPR constructs, into plant tissues. This highlights the potential of rapidly synthesized and cost-effective CDs as DNA carriers. Their ability to successfully bind plasmid DNA onto tungsten microcarriers makes them a promising tool for applications in both plant and animal science research.

However, this study focuses solely on plasmid DNA delivery and does not evaluate gene editing or gene knockdown efficiency. While the findings confirm that CDs can replace spermidine as DNA carriers, they do not establish their overall efficiency in other applications. Spermidine, though effective, is costly and challenging to synthesize in standard laboratories. CDs, therefore, offer a practical and accessible alternative. To fully validate their efficiency and utility, further studies are needed to explore their performance in gene editing and gene knockdown applications.

In the next investigation, we aimed to assess the potential of CDs for gene delivery into soybean embryos using a biolistic delivery system. This study employed genetic transformation via nanoparticle-mediated particle bombardment of soybean, coupled with a regeneration system utilizing embryogenic axes. The selection of explants was based on their straightforward extraction process from overnight-soaked soybean seeds and the high proliferation rate of meristems, which resulted in rapid shoot regeneration (Liu et al., 2004).

**FIGURE 10:**
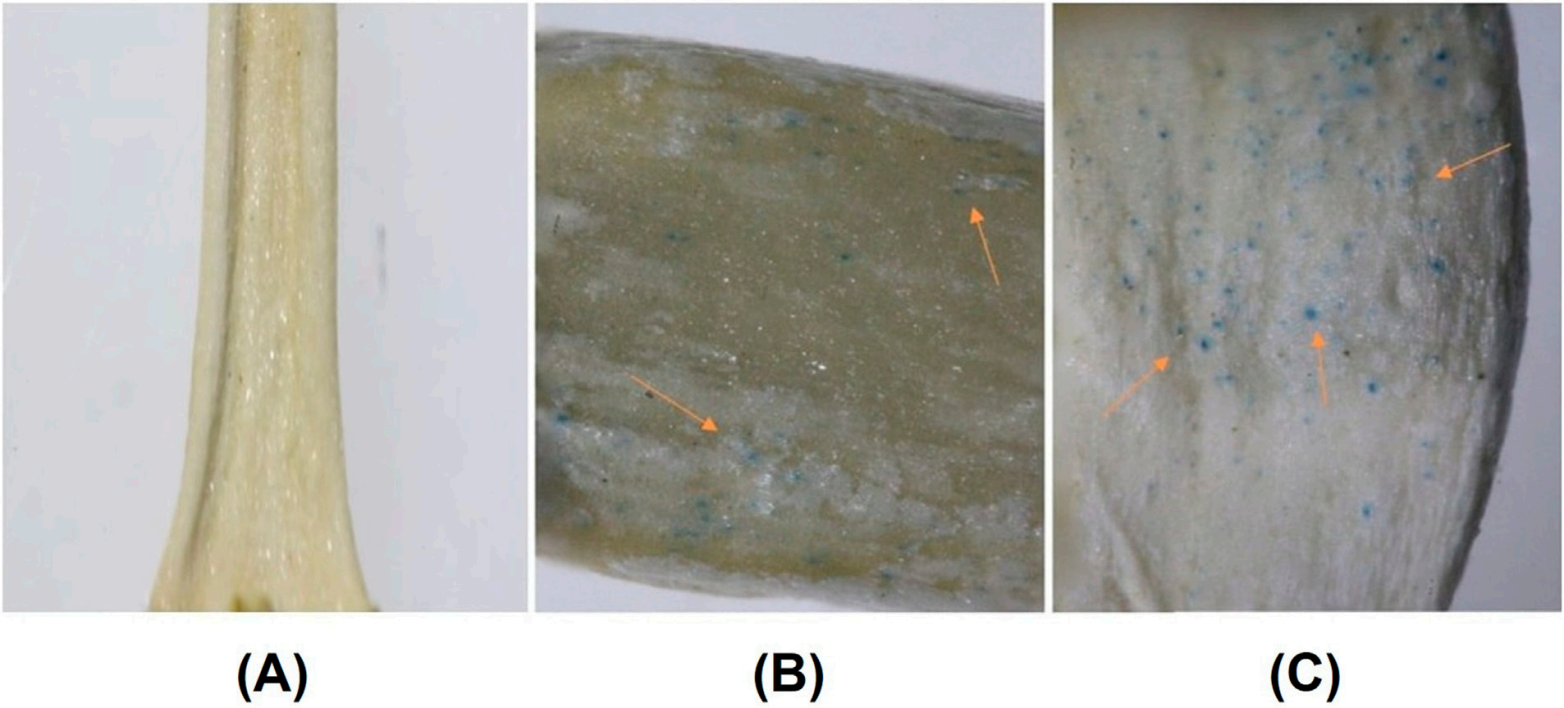
**(A)** Absence of GUS gene expression in soybean embryos where neither spermidine nor carbon dots were used as binding agents to coat the DNA with tungsten particles prior to bombardment. **(B)** GUS expression in soybean embryos following positive control spermidine-mediated gene delivery, with arrows indicating regions of GUS reporter gene expression. **(C)** GUS expression in soybean embryos following carbon dot-mediated gene delivery, with arrows indicating regions of GUS reporter gene expression.

For this purpose, soybean embryos were initially extracted from William 82 seeds. Tungsten microparticle-coated plasmid DNA/CDs were prepared and bombarded into the soybean embryos following a previously established protocol. The bombarded embryos were placed in shoot induction media for 24 h in the dark and subsequently transferred to a culture medium for 7 days. Following this period, the regeneration efficiency of the explants remained consistent between the CDs and spermidine treatments, indicating that carbon dots do not influence the regeneration efficiency, either positively or negatively, compared to spermidine, which has already been demonstrated to be safe with no adverse impact. To evaluate the gene expression in cultured embryos, histochemical analysis was performed, followed by an overnight treatment with 95% ethanol to remove chlorophyll. The embryos were subsequently imaged using a stereo microscope for detailed examination. Microscopy analysis revealed increased GUS expression in 20% of embryos bombarded with carbon dot-mediated delivery compared to spermidine condition (Figures 10B, C), whereas the absence of GUS gene expression was observed in the control group, where neither carbon dots nor spermidine were used as binding agents between the DNA and tungsten particles (Figure 10A). Compared to plant species like tobacco, wheat, and rice, soybeans exhibit significantly lower transformation efficiency and transgenic line production capabilities. However, the enhanced detection of GUS expression in 20% of developing soybean embryos highlights the potential of carbon CDs to deliver genetic material and achieve stable genetic transformation in reproductive tissues. Future optimizations in the transformation protocol, along with the use of plasmids containing desirable promoters for delivering gene-editing constructs, could substantially improve transformation efficiency in soybeans. The enhanced detection of GUS expression in the developing soybean embryos demonstrated the capability of CDs to deliver genetic material and achieve stable genetic transformation in reproductive tissues. The outcome of this experiment clearly illustrated that CDs can be used as an effective replacement for spermidine as a DNA carrier to bind plasmid DNA onto tungsten microcarriers. The special surface properties of CDs, such as the presence of various functional groups, enhance their interaction with plant cell membranes. This, in turn, has the potential to improve the absorption and incorporation of DNA into the plant genome compared to spermidine.

## Conclusion

We have demonstrated the potential of citric acid/β-alanine carbon dots as innovative nanocarriers for delivering functional DNA into various plant tissues. This study evaluated and confirmed the plasmid DNA binding efficiency of our carbon dots, a crucial factor to consider before initiating gene delivery experiments. Additionally, we showed the capability of CDs to internalize into onion epidermal cells, thereby proving their ability to penetrate plant tissues. In our investigation of carbon dot-mediated plasmid DNA delivery, we demonstrated that CDs could act as effective DNA carriers for delivering functional DNA into diverse plant tissues, including leaves, protoplasts, root cells, embryos, and epidermal cells. Various gene insertion techniques were employed, such as infiltration, passive diffusion, cellular uptake, and particle bombardment using a gene gun. Furthermore, we established that CDs could serve as an alternative to spermidine for binding plasmid DNA to tungsten microparticles during particle bombardment experiments. We also showcased the efficiency of our CDs in delivering plasmids up to 17 kb in size, including CRISPR constructs, into plant tissues. Overall, we demonstrated the rapid synthesis and cost-effectiveness of carbon dots, which can be prepared in approximately 5 minutes using only 1 g of citric acid, 0.9 g of β-alanine, and a microwave, which are commonly available in laboratories. The minimum preparation volume of these CDs is 10 mL, and only a few microliters are required per plasmid delivery experiment. These carbon dots can be stored at room temperature and used for future experiments by vortexing and sonicating for 30 s before use. Since the carbon dot synthesis method is straightforward, low-cost, rapid, and requires minimal reaction conditions, it offers significant advantages for large-scale production. Additionally, this method does not require specialized instruments for preparation, making it accessible and cost-effective. Unlike other methods that rely on functionalization with potentially risky agents such as polyethyleneimine (PEI) in some conditions, this approach avoids such concerns, ensuring a safer synthesis process. Further research is required to determine the maximum plasmid size that can be effectively delivered using these carbon dots. Additionally, it is essential to assess the potential application of these carbon dots as DNA carriers in CRISPR-Cas9-mediated gene editing experiments and for the delivery of double-stranded RNA (dsRNA) and small interfering RNA (siRNA) for gene knockdown in different plant tissues using different insertion techniques to evaluate whether the CDs show any preference for specific plant tissues or cell types and gene insertion method in different crop species.

## Data Availability

The original contributions presented in the study are included in the article/supplementary material, further inquiries can be directed to the corresponding author.
