# Correction: DNA delivery into plant tissues using carbon dots made from citric acid and β-alanine

**DOI:** 10.3389/fchem.2025.1712485

**Published:** 2025-10-16

**Authors:** Kuber Shivashakarappa, Sureshbabu Marriboina, Zeinab Yadegari, Vikas Reddy Paduri, Ritesh Sachan, Korsi Dumenyo, Ali Taheri

**Affiliations:** 1 Department of Agricultural Science and Engineering, College of Agriculture, Tennessee State University, Nashville, TN, United States; 2 Department of Life and Physical Sciences, Fisk University, Nashville, TN, United States; 3 School of Mechanical and Aerospace Engineering, Oklahoma State University, Stillwater, OK, United States

**Keywords:** DNA delivery, nanoparticle, carbon dots, gene expression, plant transformation

The citation for **Figure 8I** was erroneously written as **Figure 9I**. A correction has been made to the section **Results and discussion**, paragraph 8:

“In this next investigation, we aimed to demonstrate the delivery of DNA into protoplast and chloroplasts, subsequent transient expression of reporter genes using manufactured CDs as carriers for plasmid DNA. We obtained soybean mesophyll protoplasts from 21 day-old soybean plants through an enzymatic digestion technique. The CDs were mixed with plasmid DNA containing the GFP reporter gene and incubated at 37 °C for 30 min to facilitate binding. Subsequently, the protoplasts were cultured with the CDs-plasmid DNA complex for 24 h in a dark environment at room temperature. Confocal microscopy analysis following the incubation period showed that the CDs-plasmid DNA conjugate had penetrated the protoplasts and chloroplast membranes, localizing within the chloroplasts (**Figure 8G**). This was confirmed by monitoring the autofluorescence of chlorophyll and the combined image of GFP expression and chlorophyll autofluorescence, which exhibited the complete co-localization of GFP and the chloroplasts (**Figure 8H**). Absence of transient expression of GFP fusion proteins was noticed in the isolated soybean protoplasts and chloroplasts following incubation with the solution containing plasmid DNA alone (**Figure 8F**). The expression of the reporter gene was quantified by measuring mean fluorescence intensity following plasmid delivery with CDs as a carrier and without a carrier. In protoplasts, the mean fluorescence intensity of GFP was 83.9 ± 5.03, whereas no GFP expression was detected in the control condition where no carrier was used (**Figure 8I**). Therefore, our CDs-mediated functional DNA delivery system allows for the quick and passive diffusion of plasmid DNA into protoplasts, resulting in efficient transgenic expression without any noticeable negative impact on protoplast viability. This study demonstrates the potential of CDs as effective carriers for functional DNA delivery into chloroplasts.”

The original article has been updated.

